# Supplementary material for: Environmental inactivation and irrigation-mediated regrowth of Escherichia coli O157:H7 on romaine lettuce when inoculated in a fecal slurry matrix
Source: PeerJ. 2019 Mar 8;7:e6591. doi: 10.7717/peerj.6591 (PMC6410689; doi:10.7717/peerj.6591)
Supplement: Supplemental Information 2 — a Data collected from a HOBO weather station housed within the field site and used for regression analysis. b Data collected from a nearby CIMIS station housed at the field site and included as a comparison to conditions measured at the station used for analysis. [file peerj-07-6591-s002.docx]

**Table S1.** *Summary of weather statistics recorded by the California Irrigation Management Information System (CIMIS) and HOBO weather stations over a 236-h field trial in central coastal California, 2012.*

| **Trial** |  | **Air**  **Temp**  **(**$\boldsymbol{℃}$**)** | **Relative**  **Humidity (%)** | **Leaf Wetness** ^a^ **(%)** | **Soil**  **Temp**  **(**$\boldsymbol{℃}$**)** | **Solar**  **Radiation (W/sq.m)** | **Wind Speed (m/s)** | **Wind Direction**  **(°)** | **Vapor Pressure (kPa)** | **Dew**  **Point**  **(**$\boldsymbol{℃}$**)** |
| --- | --- | --- | --- | --- | --- | --- | --- | --- | --- | --- |
| **July 2012**  **Station 116** | **Min** | 10.7 | 74 | 3.5 | 19.8 | 0 | 0.9 | 72 | 1.4 | 10.7 |
|  | **Ave** | 13.4 | 94 | 45.8 | 20.9 | 201 | 2.8 | 250 | 1.7 | 12.3 |
|  | **Med** | 13.0 | 100 | 32.9 | 20.9 | 23 | 2.5 | 265 | 1.6 | 12.3 |
|  | **Max** | 17.8 | 100 | 100 | 21.7 | 939 | 5.2 | 332 | 2.2 | 14.7 |
|  | **SD** | 1.8 | 8.0 | 40.4 | 0.5 | 294 | 1.2 | 47 | 0.3 | 0.8 |
| **Oct 2012**  **Station 116** | **Min** | 5.9 | 18 | 2.9 | 11.9 | 0 | 0.9 | 2 | 0.7 | 2.7 |
|  | **Ave** | 14.5 | 80 | 46.1 | 16.7 | 140 | 3 | 194 | 1.3 | 10.6 |
|  | **Med** | 13.9 | 83 | 31.8 | 16.8 | 0 | 2.7 | 193 | 1.5 | 10.1 |
|  | **Max** | 29.3 | 100 | 100 | 17.8 | 679 | 7.6 | 356 | 2 | 17.5 |
|  | **SD** | 4.4 | 16 | 40.7 | 0.7 | 208 | 1.5 | 87 | 0.3 | 3.3 |
| **July 2012** ^b^  **Station 89** | **Min** | 10.3 | 58 | 3.5 | 24.5 | 0 | 0.4 | 8 | 1.2 | 9.6 |
|  | **Ave** | 14.4 | 86 | 45.8 | 26.5 | 256 | 1.7 | 298 | 1.4 | 11.9 |
|  | **Med** | 13.5 | 92 | 32.9 | 26.5 | 53.5 | 0.4 | 319 | 1.4 | 11.9 |
|  | **Max** | 22.5 | 98 | 100 | 27.6 | 928 | 7.2 | 355 | 1.6 | 14.2 |
|  | **SD** | 3.0 | 12 | 40.4 | 0.8 | 336 | 2.2 | 64 | 0.1 | 0.9 |

^a^ *Data collected from a HOBO weather station housed within the field site and used for regression analysis*

^b^ *Data collected from a nearby CIMIS station housed at the field site and included as a comparison to conditions measured at the station used for analysis*
